# Stable Two-Dimensional Conductance Switch of Polyaniline Molecule Connecting to Graphene Nanoribbons

**DOI:** 10.1038/srep05976

**Published:** 2014-08-07

**Authors:** Zhi-Qiang Fan, Ke-Qiu Chen

**Affiliations:** 1 School of Physics and Electronic Science, Changsha University of Science and Technology, Changsha 410004, People's Republic of China; 2 Department of Applied Physics, Hunan University, Changsha 410082, People's Republic of China

## Abstract

Incorporating the characteristics of the single-layer graphene nanoribbon and the polyaniline molecule, we theoretically design a two-dimensional molecular device and investigate its transport properties by applying nonequilibrium Green's functions in combination with density-functional theory. The calculated results reveal that the arrangements of frontier molecular orbitals and the energy gap between the HOMO and the LUMO of an isolated polyaniline molecule are different between its two isolable states: full reduced leucoemeraldine base and full oxidized pernigraniline base. When a polyaniline molecule connects to two graphene nanoribbons as a two-dimensional molecular device, the conductance of its full oxidized pernigraniline base is much higher than the conductance of its full reduced leucoemeraldine base. The switch ratios of two bases' currents almost maintain a constant value before 0.8 V. In other word, the conductance switch behavior in our device is stable in a big bias region which makes it have a broader application in future logic and memory devices.

Graphene, one of the allotropes of elemental carbon, is a two-dimensional nanomaterial that has between one and ten layers of sp^2^-hybridized carbon atoms arranged into a honeycomb lattice. This new material was first obtained by micro-mechanical cleavage in 2004^1^. It can be considered as the parental compound of other carbon allotropes: fullerenes, carbon nanotubes, or graphite^2^. As compared with other flat materials, graphene has demonstrated several exceptional properties such as high electron mobility at room temperature (2.5 × 10^5^ cm^2^/Vs)^3^, exceptional thermal conductivity (5 × 10^3^ W/mK)^4^, and superior mechanical properties with Young's modulus of 1 TPa^5^. Moreover, a number of fascinating electronic properties of the graphene including an ambipolar electric field effect along with ballistic conduction of charge carriers^1^, a quantum Hall effect at room temperature^6^,^7^, tunable band gap^8^, a field effect transistor^9^,^10^, the negative differential resistance behavior^11^,^12^, rectifying behavior^13^,^14^, and spin polarized electron transport behavior^15^,^16^ have been found which make it attract more and more attention.

Because of its superior electronic, thermal, and mechanical properties, the graphene has been considered as a promising next-generation conducting material to replace traditional electrode materials in electrical and optical devices in order to reduce the size of the device. So far, the researches on single-layer graphene nanoribbons (GNRs) as a two-dimensional electrode have received increasing attention in experiment^17^,^18^,^19^ and in theory^20^,^21^,^22^,^23^. It is known, GNRs can also be either metallic or semiconducting depending on the crystallographic direction of the ribbon axis. It has been predicted that GNRs with zigzag edges are all metallic. All armchair GNRs are semiconductors with different energy gaps as a function of their width. Because the switch behavior of polyaniline junction was first found in the Au-polyaniline-Au devices, we investigate the electronic transport property of a molecular device consisting of one polyaniline molecule and two single-layer zigzag GNRs.

The polyaniline molecule has received considerable attention in recent years due to the controllable electrical conductivity^24^. The structures of the polyaniline molecule exist as “salts”or “bases” in two isolable states: full reduced leucoemeraldine base (LB) and full oxidized pernigraniline base (PB). The conductivities of two isolable states are different and can be altered reversibly by oxidation (ox.) or reduction (red.)^25^,^26^. The calculated results show that the conductance of full oxidized pernigraniline base is much higher than that of full reduced leucoemeraldine base when a polyaniline molecule connects to two zigzag GNRs as a molecular device. The switch ratios of the two bases' currents are stable in a big bias region. Therefore, the role of the zigzag GNR on the transport properties of the polyaniline molecule is same to the gold electrode.

## Results

**Figure 1:**
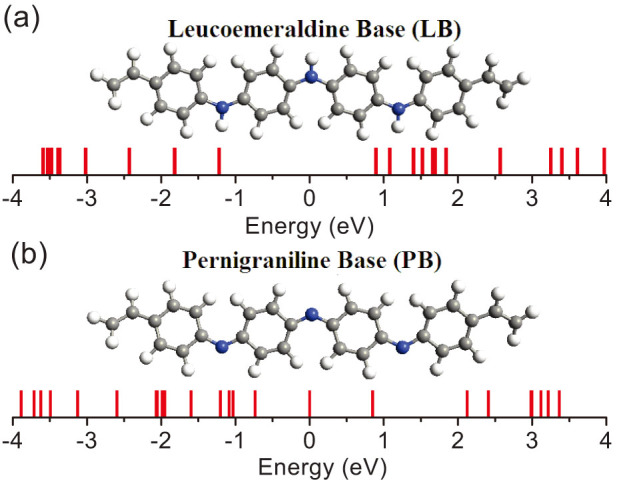
The frontier molecular orbitals of an polyaniline at (a) the leucoemeraldine base and (b) the pernigraniline base.

Firstly, we show the molecular orbitals of an isolated polyaniline at the leucoemeraldine base and the pernigraniline base in Fig. 1. For simplicity, these two bases are denoted as LB and PB, respectively. It is well known, the electronic transport properties of a molecule are directly relevant to the arrangement of its frontier orbitals. In Fig. 1(a), the highest occupied molecular orbital (HOMO) and the lowest unoccupied molecular orbital (LUMO) of LB nearly locate on two sides of the zero energy symmetrically. The HLG (energy gap between the HOMO and the LUMO) is very big (about 2 eV). For PB, the HOMO changes to −0.7 eV and the LUMO changes to 0.02 eV, see Fig. 1(b). Consequently, the removing of hydrogen atoms from nitrogen atoms reduces the HLG to 0.72 eV. As a result, the electrical conductivities of these two bases are different from each other.

**Figure 2:**
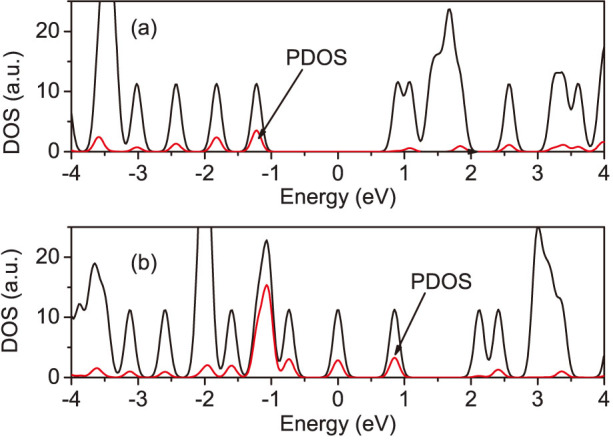
The density of states of an isolated polyaniline at (a) the leucoemeraldine base and (b) the pernigraniline base (black solid lines). The arrows indicate the projected density of states for three nitrogen atoms (red solid lines).

To identify the nature of molecular states, we give the density of states (DOS) of a polyaniline molecule and the projected density of states (PDOS) for three nitrogen atoms in Fig. 2. In Fig. 2(a), the peaks of the PDOS spectrum for three nitrogen atoms are very short. So, the nitrogen atoms of an isolated polyaniline molecule on the leucoemeraldine base have less contribution to the molecular DOS. When the polyaniline molecule is full oxidized, the DOS spectrum of PB moves to higher energy as compared with the DOS spectrum of LB. More importantly, a new broad and high DOS peak presents at −1.1 eV. The corresponding peak of PDOS of nitrogen atoms is also broad and high. It is several times larger than other peaks. That means this new DOS peak at −1.1 eV is mainly induced by these three nitrogen atoms. So, the removing of hydrogen atoms from nitrogen atoms alters the electronic properties of polyaniline molecule effectively.

**Figure 3:**
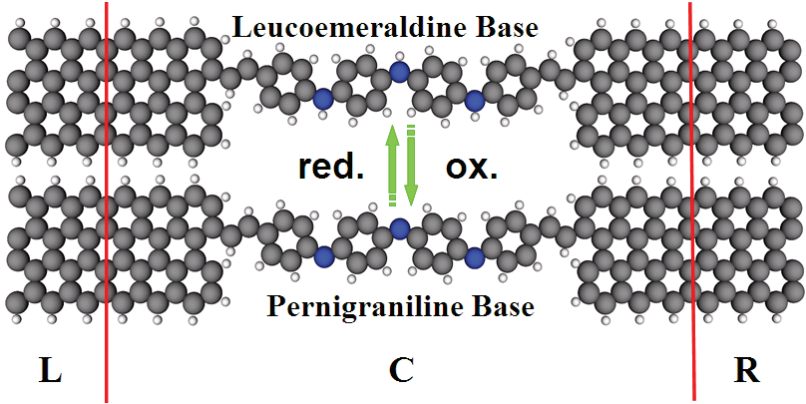
Sketch of our models: The top and bottom devices are the molecule on the full reduced leucoemeraldine base and the full oxidized pernigraniline base, respectively. When the molecule is full oxidized, the three hydrogen atoms are removed from the nitrogen atoms.

Next, we connect a polyaniline molecule to two single-layer zigzag graphene nanoribbons as a device and study its electronic transport properties. The molecular devices are illustrated in Fig. 3, which is divided into three regions: left electrode (L), right electrode (R), and the central scattering region (C). The central scattering region contains a polyaniline molecule and a portion of the graphene electrodes, which is included in the self-consistent cycle to take into account the molecule-electrode coupling and electrode screening effect. In our device, the polyaniline molecule mainly consists of the carbon atom and the molecule connects to the zigzag GNR electrode through the C-C single bond. So, the position of attachment to the electrode has little effect on the transport properties of our devices. To improve the calculation efficiency, the width of the zigzag GNR just covers the polyaniline molecule. Test calculations show that the very similar transmission spectra are obtained by using wider zigzag GNR as the electrode. For simplicity, the single-layer full reduced leucoemeraldine base and the full oxidized pernigraniline base molecular devices are named as SL and SP.

**Figure 4:**
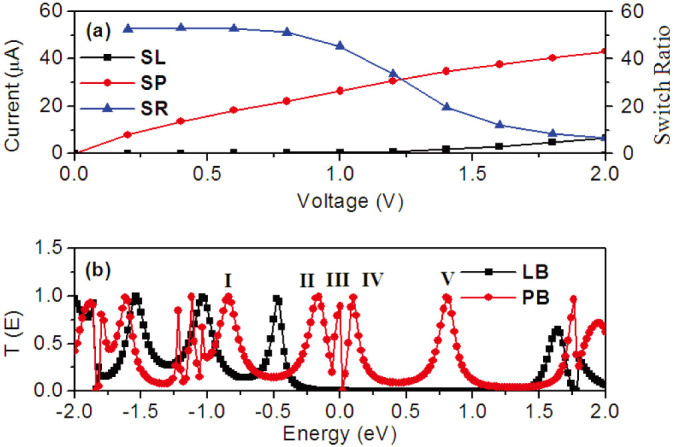
(a) Current-voltage curves of device SL and device SP, and switch ratio (SR = I_SP_/I_SL_) changes with the positive bias. (b) The transmission spectra of device SL and device SP at zero bias. The energy origin is set to be the Fermi energy of the graphene electrode.

Fig. 4(a) shows the current-voltage characteristics of single-layer devices SL and SP. One can see that the currents of SL are tiny in bias region from 0 V to 1.2 V. That means the conductance of the polyaniline molecular device on full reduced leucoemeraldine base is very small. Unlike the devices SL, the currents of SP increase rapidly with bias voltages from 0 V to 2 V. Therefore, there is a switch from the low conductance (OFF state) to high conductance (ON state) when a polyaniline molecule changes between two bases. This switch behavior can be clearly reflected by the current switch ratios (SR) as a function of the bias. Two important features in the evolution of SR are clearly visible: (1) the SR is stable before 0.8 V and after 1.6 V; (2) it falls rapidly in bias region from 1.0 V to 1.4 V. Generally, the current switch ratios of a part of molecular switches in previous reports are small and also oscillated with applied bias^27^,^28^,^29^,^30^. So, the switch behaviors are easily affected by the bias voltage. However, the switch behavior in our device is obvious and stable in a big bias region, which makes it have a broader application in future logic and memory devices.

In order to explore the difference on conductance of SL and SP, their transmission spectra at the equilibrium state are shown in Fig. 4(b). It is well known, the transmission coefficient is the most intuitive representation of quantum transport behaviors of the devices. The Fermi energy level of our molecular device is set to be the Fermi energy of the graphene electrode. The alignment between the frontier molecular orbitals and the Fermi energy level of electrode plays a pivotal role in the conductance of the device. Although the Fermi energy level of electrodes is close to the HOMO of LB, it is still in the middle of the HOMO and the LUMO. There is only one transmission peak at −0.5 eV in the energy region [−1 eV, 1 eV]. The transmission coefficient in the vicinity of the Fermi level is almost zero leading to the big HLG (see Fig. 2(a)). Consequently, the frontier molecular orbitals of a polyaniline molecule on the full reduced leucoemeraldine base have less contribution to electronic transport. So, the corresponding conductance is very small. For device SP, there are five high and broad transmission peaks in the energy region [−1 eV, 1 eV]. The new high DOS peak induced by nitrogen atoms and its neighboring DOS peak around the −1 eV in Fig. 2(b) align with the Fermi energy level of electrodes very well. So, the transmission coefficients in the vicinity of the Fermi level are bigger than that of the device SL. As a result, the relevant conductance is enhanced considerably.

**Figure 5:**
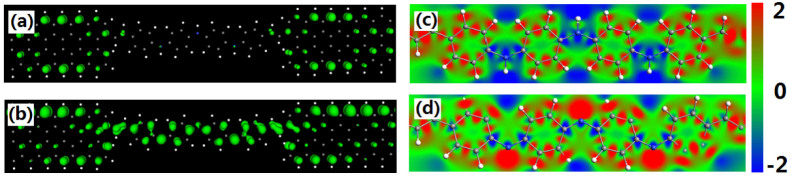
(a) and (b) The spatial LDOSs of device SL and device SP on Fermi energy. The isosurface value is 0.05. (c) and (d) The contour cut-planes of electrostatic potential for device SL and device SP at zero bias. The red and blue colors represent the high and low electrostatic potentials.

To explore the physical mechanism of the conductance switch behavior between the device SL and the devices SP, we plot their spatial local density of states (LDOS) on Fermi energy and the electrostatic potential distribution at zero bias in Fig. 5. The LDOS can tell us the contributions of every atoms to the device DOS. It had been adopted to explore the transport properties in several previous papers^31^,^32^,^33^. Fig. 5(a) shows the density of states of the device SL only located on both graphene electrodes. The central molecule has no contribution to the device's DOS. So, the transmission coefficient in the vicinity of the Fermi level is almost zero. In contrast, a substantial density of states of SP device localized at the central scattering region entirely in Fig. 5(b). The graphene electrode and the central molecule favor the electronic transport and open the transmission channels under very low bias. Furthermore, the electrostatic potential distribution also can be used to explain the huge difference of the conductance between the device SL and the devices SP. The charge transfers across the electrode-molecule interface can change the electrostatic potential of the junction effectively^34^. A crosssectional view of the electrostatic potential on molecule LB in Fig. 5(c) shows the net transfer of electrons into the molecule increases the electrostatic potentials of all carbon atoms. But the electrostatic potentials on three nitrogen atoms and their connected hydrogen atoms are very low. There are three potential well in the molecule LB. It is well known, the presence of the potential well in the molecule core will affect the charge redistribution within the molecule when an additional field is applied and impedes the flow of electrons across junction^34^,^35^,^36^. For this reason, the conductance of the device SL is very low. When the polyaniline molecule changes to full oxidized pernigraniline base, the hydrogen atoms are removed from the nitrogen atoms. The net transfer of electrons into the molecule increases the electrostatic potentials of all carbon atoms and nitrogen atoms (see Fig. 5(d)). More important, the electrostatic potentials on the carbon and nitrogen atoms are same, resulting in the average and flat electrostatic potential distribution on the entire PB molecule. Thus, the electron can easily flow through the molecule.

**Figure 6:**
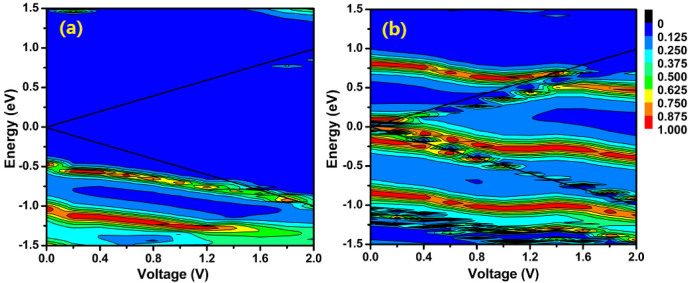
(a) and (b) The transmission coefficients of device SL and device SP as a function of the bias. The region between two solid lines is the bias window.

Moreover, the stable current switch behavior in large bias region is another unique character of our device. To explain it, we plot the transmission as a function of the energy and bias voltage in Fig. 6. As discussed above, the closest transmission peak of the device SL near the Fermi energy locates at −0.5 eV. When the forward bias was applied, this transmission peak moves to lower energy slowly and is always outside of the bias window until 1.2 V, see Fig. 6(a). So, the corresponding currents before 1.2 V are very small. When the forward bias further increases, although this transmission peak still follows the edge of the negative bias window, its tail enters the bias window to enhance the currents. In Fig. 6(b), the transmission peaks I, II, III, V of the device SP move downwardly to lower energy, but the transmission peaks IV moves upward to higher energy. The transmission peak III enters the bias window first because it aligns with the Fermi energy. Then, the transmission peak IV enters the bias window to enhance the currents. However, the transmission coefficient of this peak decreases gradually after 0.4 V. The transmission peak II moves into bias window at 0.4 V to counteract the coefficient decreases of the transmission peak IV. As a result, the total transmission coefficient in the bias window increases uniformly leading to the linear rise of the current before 0.8 V. Consequently, the SR is stable before 0.8 V in Fig. 4(a). Increasing the bias from 1.0 V to 1.4 V, one can see a remarkable decrease in the contribution of the transmission peak II on the current due to the decrease of its coefficient. There is no other transmission peak moves into bias window to counteract the loss of the coefficient. So, the SR falls rapidly in this bias region. Upon increasing the applied bias, it is seen from Fig. 6(b) that the transmission peaks V shifts into the negative bias window after 1.4 V and the transmission peaks I also shifts close to the positive bias window to enhance the current. However, the current of the device SL also increase with bias. Although the corresponding SR is stable, the value is smaller as compared with bias region [0.2 V, 0.8 V].

## Discussion

By applying density-functional theory and non-equilibrium Green's function method, we have investigated the quantum transport properties of a polyaniline molecule and two single-layer graphene nanoribbons as a two-dimensional molecular device. The arrangement of frontier molecular orbitals and the energy gap between the HOMO and the LUMO of a polyaniline molecule are different when the molecule alters among two isolable states by oxidation/reduction. A new broad and high DOS peak was induced by the three nitrogen atoms on full oxidized pernigraniline base. This suggests that the oxidation/reduction could be used to modulate the electronic structures of the polyaniline molecule. Once a polyaniline molecule connecting to two single-layer graphene nanoribbons as a two-dimensional molecular device, the calculated results show the conductance of SP device is much higher than the conductance of SL device. We demonstrate that the alignment between the Fermi energy of the electrode and the frontier molecular orbitals of two bases of the polyaniline molecule is a main reason of the conductance switch behavior in our device. The removing of hydrogen atoms on SP device enhances its spatial local density of states on the molecule and improves the electrostatic potential around the nitrogen atoms, resulting in the transfer of electrons in the junction. For SP device, the three transmission peaks in the vicinity of the Fermi level in turn come into bias window leading to the linear rise of the current. So, the current switch behavior in our device is stable in a big bias region which makes it having a broader potential in future logic and memory devices.

## Methods

The method in this paper is an *ab* initio code package based on nonequilibrium Green's functions and density functional theory^37^,^38^. The exchange-correlation potential is described by the local density approximation (LDA), which works rather well for light elements and systems where electrons are delocalized^39^. The basis set for all atoms are double-zeta plus polarization (DZP). The Hamiltonian, overlaps, and electronic densities are evaluated in a real space grid defined with a plane wave cut off of 150 Ry to achieve a balance between calculation efficiency and accuracy. The geometries are optimized until all residual force on each atom is smaller than 0.05 eV/Å. The current is calculated by using the Landauer-like formula, 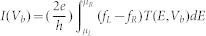, where *μ_L/R_* is the electrochemical potentials of the left and right electrodes and *f_L/R_* are the Fermi distribution function for left and right electrodes^40^,^41^. With the applied bias potential *V_b_*, the chemical potential of left electrode *μ_L_*(*V_b_*) = −*eV_b_*/2 and the chemical potential of right electrode *μ_R_*(*V_b_*) = +*eV_b_*/2. The energy region of the transmission spectrum that contributes to the current integral in the Landauer-like formula is referred to the bias window [−*V_b_*/2, + *V_b_*/2]. The transport coefficient *T*(*E*, *V_b_*) can be calculated using the well-known formula, *T*(*E*,*V_b_*) = *Tr*[Γ*_L_*(*E*)*G^R^*(*E*)Γ*_R_*(*E*)*G^A^*(*E*)], where *G^R^*(*E*) and *G^A^*(*E*) are the retarded and advanced Green's functions, respectively, and 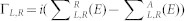 is the coupling functions of the conductor to the left and right electrodes, 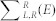 and 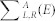 are the self-energy matrices used to include the effect of the left (right) semi-infinite electrode.

## Author Contributions

K.Q.C. performed the device design and theoretical analysis, Z.Q.F. calculated geometrical properties, electronic structures, transmission spectra, and the I-V characteristics. All the authors discussed the results and wrote the manuscript.
